# Supplementary material for: Sex Differences in Behavior and Learning Abilities in Adult Rats
Source: Life (Basel). 2023 Feb 15;13(2):547. doi: 10.3390/life13020547 (PMC9966297; doi:10.3390/life13020547)
Supplement: Supplementary file 1 [file life-13-00547-s001.zip › life-2202002-supplementary-revised.pdf]

Supplementary S1. Electroencephalographic examination in Group 2.

High voltage spike-wave discharges (SWD) in EEG are the hallmark of seizure activity in the mother WAG/Rij rat strain. 11-24 hours of EEG. This part of study was done in NEW rats from Group 2. In one male recording was not successful. In total, 6 males and 8 females of Group 2 were examined for the presence of spike-wave EEG abnormalities in their electroencephalograms (EEGs). Rats were implanted with chronic epidural electrodes for 3-channel recordings in free behavior. After a 10-14 day recovery period, rats were placed in a Plexiglas recording cage (Fig.S1). EEG was recorded during 22-24 hours (in 2 rats records were shorter due to technical problems) using a multichannel amplifier (PowerLab 4/35, LabChart 8.0 software, ADInstruments, Sydney, Australia) via a swivel contact, band-pass filtered between 0.5–200 Hz, digitized with 400 samples/second/per channel and stored in the hard disk (Fig.S1).

This part of the study was approved by the Ethics Committee of Institute of the Higher Nervous Activity and Neurophysiology of the Russian Academy of Sciences (protocol № 4 approved on 26 October 2021).

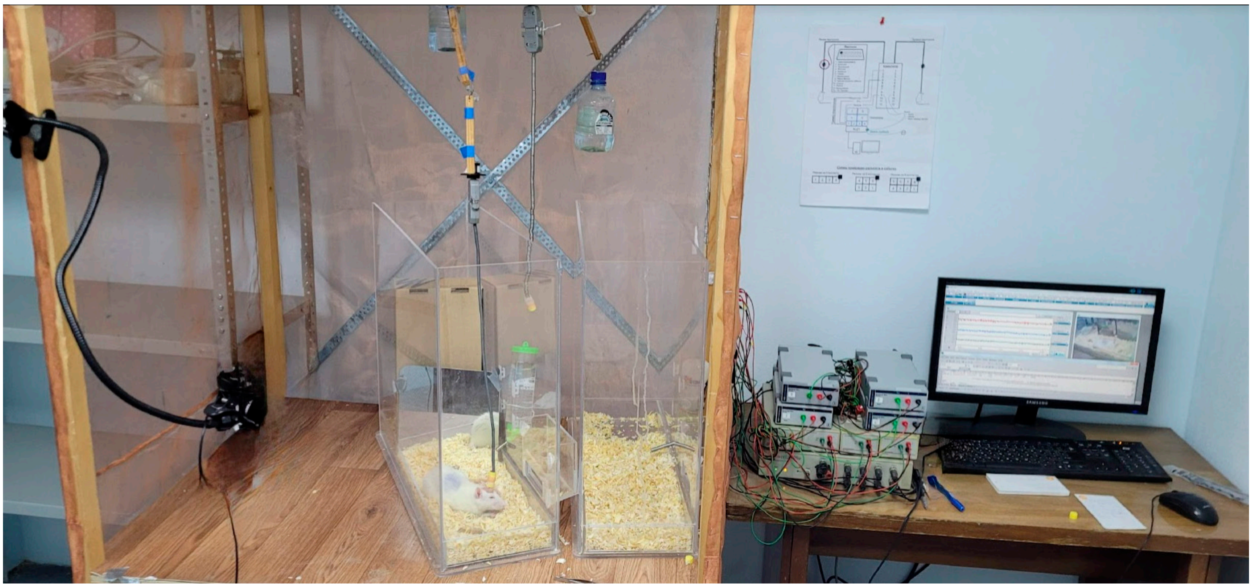

Figure S1. The photo of EEG-recording setup (PowerLab 4/35 and computer). In this particular experiment, video-EEG was recorded in a freely moving rat.

Figure S2 demonstrates regular spike-wave discharges, SWD, in EEG recorded in adult WAG/Rij rat. Typical SWD in WAG/Rij rats represents a sequence of 5-10 s high-voltage spike-wave complexes with robust onset and strong asymmetry. The amplitude of SWD usually 3-5 times exceeded the background. Spikes in SWD were very regular and repetitive. NEW rats usually expressed short-lasting SWD 2-3 s with a spindle-like envelope consisting of several spike-wave complexes (Fig. S3, the upper plot).

SWD were selected automatically using wavelet-based algorithm described in our earlier papers [1,2]. Briefly, EEG signal from the frontal EEG channel underwent a continuous wavelet transform. Wavelet power was computed in the main frequency band of SWD, W[8-10 Hz], and its 1st harmonics - W[17-20 Hz]. Threshold values for W[8-10 Hz] and W[17-20 Hz] were chosen between 100-210 and adjusted individually. In order to define the end of SWD, wavelet power was smoothed by 10-20 %.

Figure S4 demonstrates SWDs in WAG/Rij rat, which were automatically detected in the frontal EEGs (channel "EEG"), by computing wavelet power in W[8-10 Hz] and W[17-20 Hz] frequency bands with the thresholds of 170. The minimal duration of the SWD was set at 2000 ms.

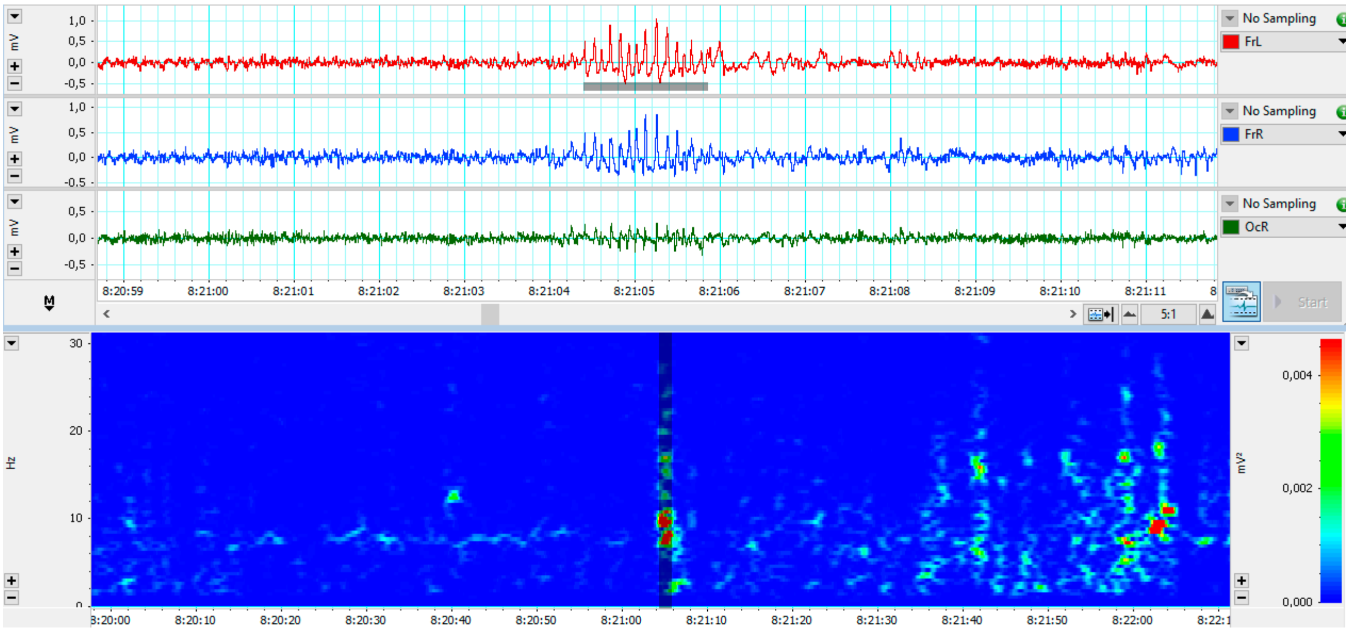

(*a*)

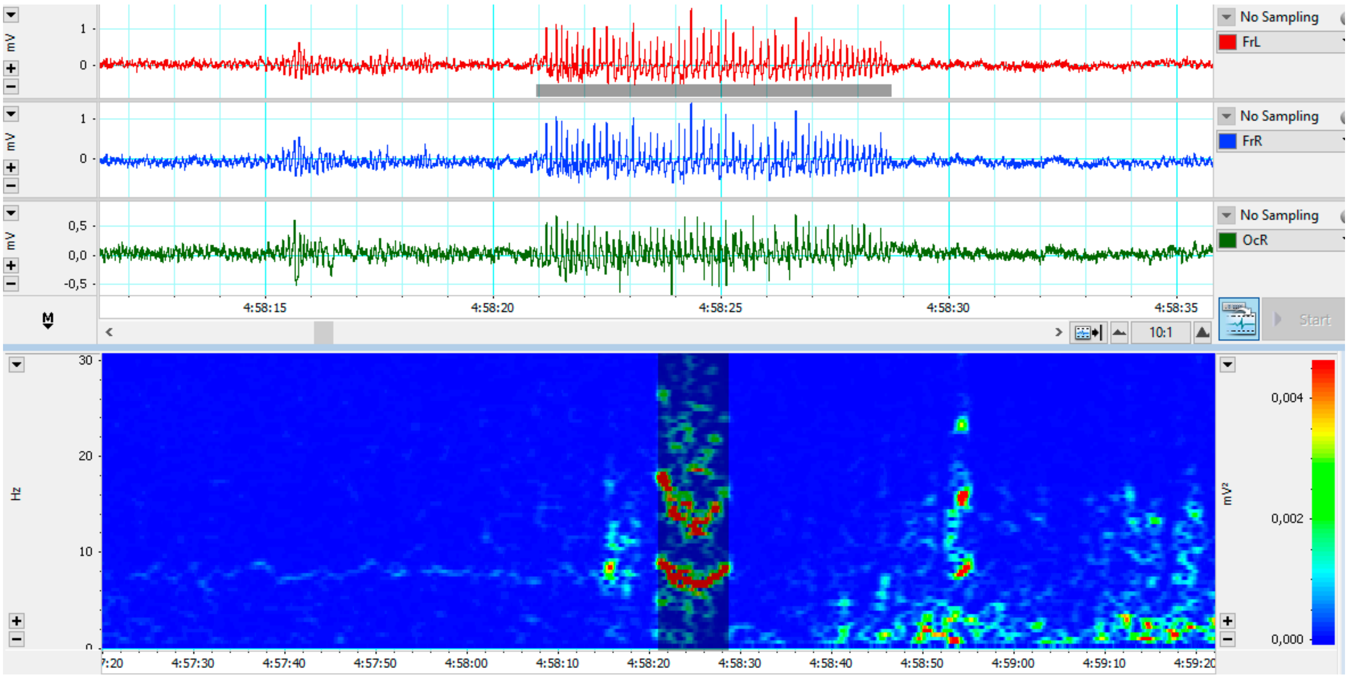

(*b*)

Figure S2. Examples of spike-wave discharges (SWD) as recorded in a freely moving WAG/Rij rat. (a) – Short-lasting SWD and (b) and (b) – fully blown SWD. Three-channel records were obtained at the frontal left and right cortex, FrL and FrR (symmetrical areas) and at the occipital right cortex, OcR and plotted in LabChart 8.0. Time is given in hh:mm:ss. The bottom plots show power spectra of the FrL (frontal left EEG channel) for frequencies 0-30 Hz, where spike-wave discharges are shaded. Spectra are obtained with Hann-window FFT 1024 size and 93.5% overlap. Note the difference in time scales of raw EEG and its spectrum. EEG time scales in (a) and (b) are the same.

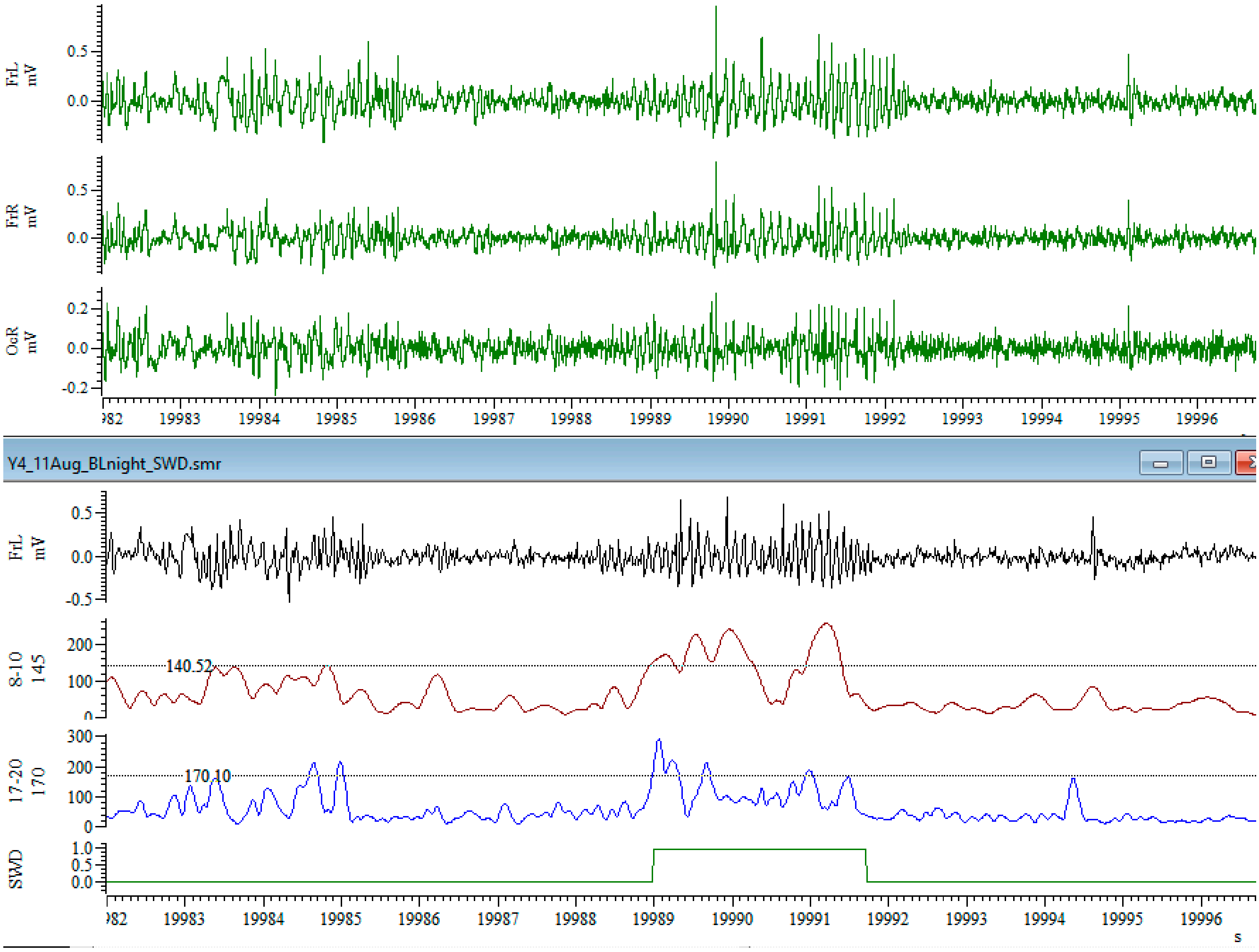

Figure S4. Short-lasting SWD as recorded with 3-channels EEG in an adult NEW rat (ID=Y4) and plotted in Spike 5. Time is given in s. The signal was recorded at the FrL and FrR (the frontal left and right cortex, symmetrical areas) and at the OcR occipital right cortex. The bottom plot shows results of automatic detection of SWD performed in FrL EEG channel; distribution of wavelet power as measured at 8-10 Hz and at 17-20 Hz - channels "W[8-10 Hz]" and "W[17-20 Hz]". The threshold level in "W[8-10 Hz]" was set at 140 and in "W[17-20 Hz]" – at 170.

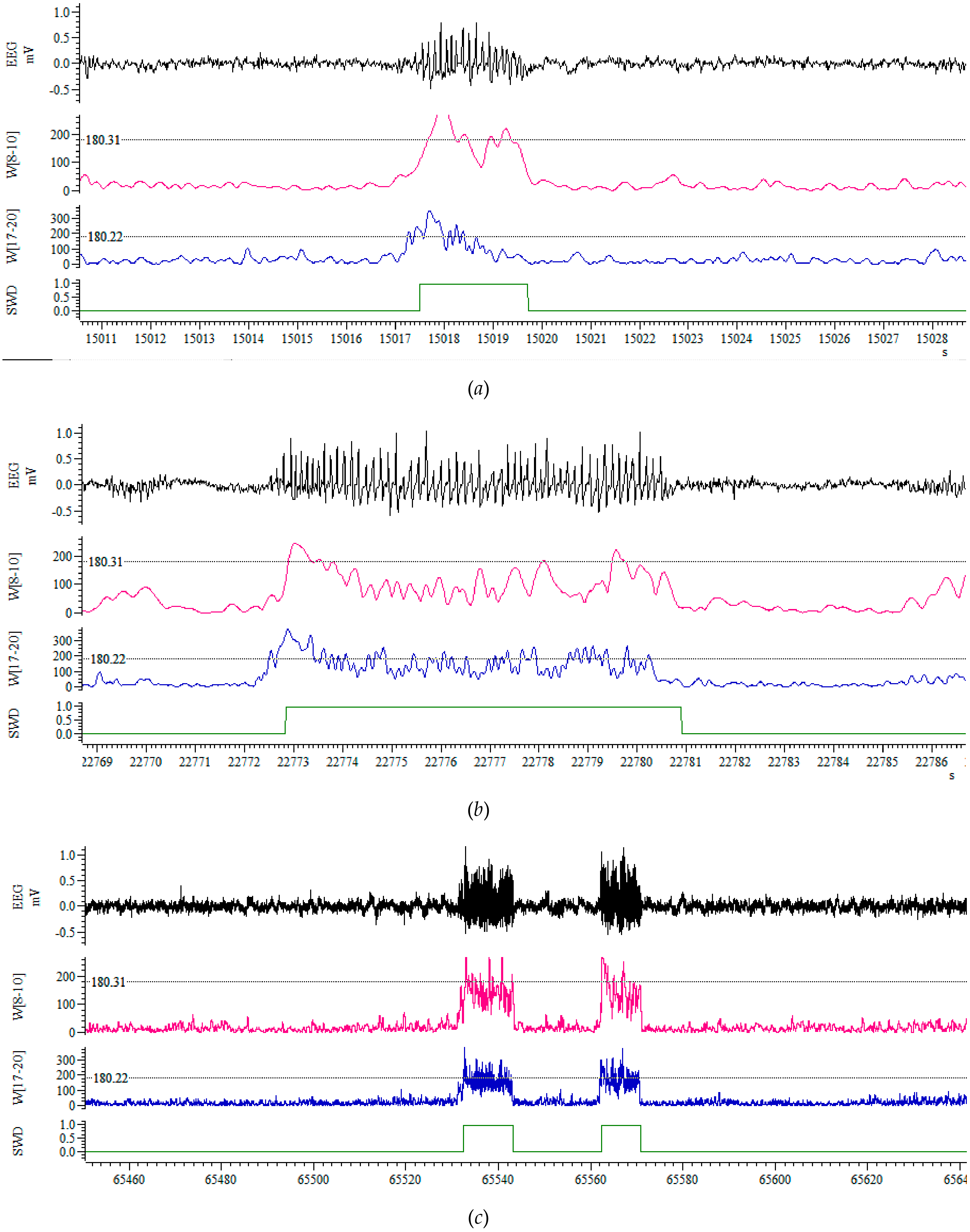

Figure S4. Details of automatic detection of spike-wave discharges (SWD) in adult WAG/Rij rat. (a) - Short-lasting SWD; (b) – a typical SWD; (c) - 192 s epoch with two SWDs. Time is given in s. The 1st channel EEG as recorded at the frontal left cortex; Channel "W[8-10 Hz]" - wavelet power was computed at 8-10 Hz; Channel "W[17-20 Hz]" - computed at 17-20 Hz. The threshold in both wavelet channels was set at 180.

Table S1. Results of automatic detection of SWD in NEW rats of Group 2.

ID	Channel	Band 1, Hz	Threshold for Band1	Band 2, Hz	Threshold for Band2	Min duration of SWD, ms	Duration of analyzed EEG, s	Total duration of SWD	Total number of SWD	SWD density s/per hour	Mean duration of SWD
Y1	FrR(1)	8-10	180	17-20	180	2000	70 400	2.2	1	0.11	2.20
Y2	FrR(1)	8-10	180	17-20	185	2000	40 000	63.2	18	5.69	3.51
Y4	FrL(0)	8-10	140	17-20	170	2000	54 950	24.1	10	1.58	2.41
Y9	FrR(1)	8-10	150	17-20	100	2000	89 450	16.0	5	0.64	3.20
Y0	FrR(1)	8-10	170	17-20	210	2000	74 600	0.0	0	0.00	
Y00	FrR(1)	8-10	170	17-20	210	2000	78 200	0.0	0	0.00	
X1	FrL(0)	8-10	180	17-20	180	2000	40 000	11.0	3	0.99	3.67
X2	FrL(0)	8-10	180	17-20	180	2000	72 400	2.3	1	0.11	2.30
X3	FrL(1)	8-10	180	17-20	180	2000	78 200	4.5	2	0.21	2.25
X4	FrL(0)	8-10	180	17-20	180	2000	74 600	25.9	11	1.25	2.35
X5	FrL(0)	8-10	180	17-20	180	2000	78 200	0	0	0	
X6	FrL(0)	8-10	180	17-20	210	2000	72 400	4.7	2	0.23	2.35
X7	FrL(0)	8-10	180	17-20	180	2000	78 200	6.5	2	0.30	3.25
X8	FrL(0)	8-10	180	17-20	180	2000	72 200	12.2	4	0.55	3.01

Individual data acquired by automatic detection of SWD are shown in Table S1. The length of the analyzed EEG varied from 40000 to 894500 s (11.1 - 24.8 hours), and it was limited by requirements of wavelet-based processing and by the quality of recorded EEG signal. In 13 rats SWD of Group 2, SWD density was scored in s/per hour, and it varied from 0 s (minimum) to 5,69 s (maximum). Rat Y2 had the highest value of seizure density: 5.69 s per hour, and the mean duration of SWD was 3.51 s. The other rats showed short-lasting immature spike-wave activity (Fig. A3).

References

1. Hramov, A.E.; Koronovskii, A. A.; Makarov, V.A.; Maksimenko, V.A.; Pavlov, A.N.; Sitnikova, E. *Wavelets in Neuroscience*; Springer Series in Synergetics; Springer International Publishing: Cham, 2021; ISBN 978-3-030-75991-9.

2. Sitnikova, E.; Smirnov, K. Active Avoidance Learning in WAG/Rij Rats with Genetic Predisposition to Absence Epilepsy. *Brain Res. Bull.* **2020**, *165*, 198–208, doi:10.1016/j.brainresbull.2020.10.007.

Supplementary S2. Results of behavioral tests in Group 1. Individual data and cluster analysis

Table S1. Clustering of rats (Group 1) according to their weight dynamics in the sucrose preference test. M1 and M1 – male cluster 1 and 2. F1 and F2 - male cluster 1 and 2.

N	ID	Sex	Weight Cluster	Weight, g			Weight dynamics, g	
				0 Day	1 Day	2 Day	1 Day	2 Day
1	Y0_1	male	M1	418	423	420	5	-3
2	Y0_2	male	M1	330	337	337	7	0
3	Y0_3	male	M1	402	408	406	6	-2
4	Y0_4	male	M1	382	386	383	4	-3
5	Y0_5	male	M1	407	410	410	3	0
6	Y00_1	male	M1	358	364	367	6	3
7	Y00_2	male	M2	330	331	334	1	3
8	Y00_3	male	M2	419	421	431	2	10
9	Y00_4	male	M2	390	390	395	0	5
10	Y00_5	male	M2	410	406	410	-4	4
11	Y1_1	male	M2	358	353	357	-5	4
12	Y1_2	male	M1	382	384	384	2	0
13	Y1_3	male	M2	364	365	372	1	7
14	Y1_4	male	M1	358	360	360	2	0
15	Y1_5	male	M2	390	393	400	3	7
16	Y1_6	male	M1	376	379	382	3	3
17	Y1_7	male	M1	361	362	362	1	0
18	Y2_1	male	M2	394	389	395	-5	6
19	Y2_2	male	M1	355	369	365	14	-4
20	Y2_3	male	M2	410	406	412	-4	6
21	Y2_5	male	M2	345	343	344	-2	1
22	Y3_1	male	M1	365	370	371	5	1
23	Y3_2	male	M2	377	374	379	-3	5
24	Y3_3	male	M2	365	363	369	-2	6
1	X0_1	female	F1	220	216	213	-4	-3
2	X0_2	female	F1	229	227	226	-2	-1
3	X0_3	female	F2	232	222	218	-10	-4
4	X0_4	female	F2	227	220	224	-7	4
5	X1_1	female	F1	207	206	202	-1	-4
6	X1_2	female	F2	215	208	205	-7	-3
7	X2_1	female	F1	224	221	224	-3	3
8	X2_2	female	F2	209	204	208	-5	4
9	X2_3	female	F1	216	214	213	-2	-1
10	X3_1	female	F1	209	208	209	-1	1
11	X3_2	female	F1	210	212	213	2	1

Table S2. Clustering of rats (Group 1) in behavioral tests.
The sucrose preference test, weight dynamics clusters: M1 and M1 – male cluster 1 and 2. F1 and F2 - male cluster 1 and 2.

The von Frey test, clusters: L – low 50% threshould; H – high 50% threshould

N	ID	Sex	Sucrose pref. weight cluster	T-maze scores	T-maze code	von Frey cluster	von Frey 50% threshould
1	Y0_1	male	M1	no preference	RRL0L	L	0.00126
2	Y0_2	male	M1	R	LRRR0	L	0.00424
3	Y0_3	male	M1	no preference	LRLR0	H	0.01453
4	Y0_4	male	M1	R	RLRRR	H	0.01345
5	Y0_5	male	M1	L	RLLLR	H	0.01016
6	Y00_1	male	M1	L	LLR00	L	0.00174
7	Y00_2	male	M2	R	RRLR0	H	0.0489
8	Y00_3	male	M2	passive	LL000	L	0.00302
9	Y00_4	male	M2	R	RLRRR	L	0.00503
10	Y00_5	male	M2	R	RLR00	L	0.00541
11	Y1_1	male	M2	no preference	LRL0R	L	0.00092
12	Y1_2	male	M1	R	RLR00	H	0.035
13	Y1_3	male	M2	no preference	RLR0L	L	0.00745
14	Y1_4	male	M1	no preference	RLL0R	H	0.01161
15	Y1_5	male	M2	no preference	LLR0R	L	0.00807
16	Y1_6	male	M1	no preference	LR0LR	H	0.035
17	Y1_7	male	M1	no preference	LR0LR	H	0.035
18	Y2_1	male	M2	no preference	RLR0L	L	0.00503
19	Y2_2	male	M1	passive	R00L0	H	0.01453
20	Y2_3	male	M1	passive	0000L	L	0.00697
21	Y2_5	male	M2	no preference	RLR0L	L	0.00218
22	Y3_1	male	M1	R	RLRRL	L	0.00807
23	Y3_2	male	M2	L	RLL00	H	0.035
24	Y3_3	male	M2	L	LRLLR	H	0.01341
1	X0_1	female	F1	no preference	RLLR0	L	0.00872
2	X0_2	female	F1	no preference	LRLR0	L	0.00503
3	X0_3	female	F2	L	LRLLR	L	0.00946
4	X0_4	female	F2	R	LRR00	H	0.01453
5	X1_1	female	F1	no preference	LRRL0	L	0.00568
6	X1_2	female	F2	R	RLRLR	L	0.00609
7	X2_1	female	F1	L	RLL00	L	0.00609
8	X2_2	female	F2	L	LLL00	L	0.00122
9	X2_3	female	F1	no preference	LRRL0	L	0.00783
10	X3_1	female	F1	R	RR0LR	L	0.00218
11	X3_2	female	F1	no preference	LRR00	L	0.00745
